# *Lactobacillus* lysates protect oral epithelial cells from pathogen-associated damage, increase secretion of pro-inflammatory cytokines and enhance barrier integrity

**DOI:** 10.1038/s41598-025-86914-y

**Published:** 2025-02-18

**Authors:** Steven D. Mercer, Christopher Doherty, Gurdeep Singh, Thomas Willmott, Tanaporn Cheesapcharoen, Rawee Teanpaisan, Catherine O’Neill, Ruth G. Ledder, Andrew J. McBain

**Affiliations:** 1 Division of Pharmacy and Optometry, School of Health Sciences, Faculty of Biology, Medicine and Health, The University of Manchester, Manchester, UK; 2 Division of Musculoskeletal and Dermatological Sciences, School of Health Sciences, Faculty of Biology, Medicine and Health, The University of Manchester, Manchester, UK; 3 Institute of Infection, Veterinary and Ecological Sciences, Clinical Infection, Microbiology & Immunology, University of Liverpool, Liverpool, UK; 4 Department of Conservative Dentistry, Faculty of Dentistry, Prince of Songkla University, Hat- Yai, Thailand; 5 Medical Science Research and Innovation Institute, Prince of Songkla University, Hat-Yai, Thailand; 6 Lydia Becker Institute of Immunology and Inflammation, Faculty of Biology, Medicine and Health, The University of Manchester, Manchester, UK

**Keywords:** Probiotics, Postbiotics, Oral-disease, Oral-health, Periodontitis, Applied microbiology, Bacteria, Pathogens, Experimental models of disease

## Abstract

Periodontitis is a chronic gum disease characterised by inflammation and the loss of bone. We have explored the potential prophylactic effects of lysates from four *Lactobacillus* strains against the toxic effects of three periodontal pathogens *(Porphyromonas gingivalis*,* Fusobacterium nucleatum*, and *Aggregatibacter actinomycetemcomitans*). TR146 oral epithelial cells were pre-treated with *Lactobacillus* lysates (*L. rhamnosus -* GG, *L. rhamnosus -* SD11, *L. reuteri* and *L. plantarum*) and then challenged with pathogenic material (live cells, lysates, or supernatants). Cytokine analysis was performed on supernatants of cells treated with probiotic lysates from 1.5 h to 24 h. Effects of probiotic lysates on re-epithelialisation were determined using keratinocyte scratch assays, monitoring both migration and proliferation. Epithelial barrier function was observed after lysate addition by trans-epithelial electrical resistance (TEER) and by quantifying claudin-1 expression. Treatment of host cells with *Lactobacillus* lysates before pathogen exposure conferred significant protection against viability loss. Although extended pre-treatment did not generally increase protection, against live *Aggregatibacter actinomycetemcomitans*, significant increases in viability were seen after 24 h of pre-treatment for GG, SD11 and *L. plantarum.* Pro-inflammatory cytokines TNF-α, IP-10, IL-6, and IL-8 increased significantly with extended probiotic treatment, while IL-1β and IL-1α secretion significantly increased but remained constant over time. Secretion of the growth-promoting cytokine TGF-β increased after 3 h of treatment, however no increases in the regulatory cytokine IL-10 were recorded. Only exposure to SD11 significantly enhanced re-epithelialisation, TEER and claudin-1 expression while GG increased TEER but decreased claudin-1 expression. *L. plantarum* significantly inhibited re-epithelialisation but did not impact TEER or claudin-1 expression. All lysates significantly improved TEER in the presence of pathogenic material, demonstrating a protective effect on barrier function.

**Supplementary Information:**

The online version contains supplementary material available at 10.1038/s41598-025-86914-y.

## Introduction

The World Health Organisation (WHO) estimates that 3.5 billion people were affected by oral diseases worldwide in 2022, with periodontitis accounting for 19% of these cases^1^. Periodontitis is a chronic gum disease associated with host-mediated inflammation and the loss of key structural oral elements, including bone^2^. It is often characterised by the increased abundance of common periodontal pathogens. In the UK, NHS dental care is currently facing significant challenges, contributing to deteriorating oral health^3^. As access to healthcare for many, especially those in the developing world, is challenging; paired with an increased incidence of periodontitis^1^, there is a pressing need for new sustainable treatments to combat oral disease.

The term probiotic refers to live microorganisms administered to exert positive effects on a host^4,5^. Recent studies have considered the enteral and topical application of probiotics in skin care to enhance barrier integrity and treat conditions such as eczema^6,7^. Additionally, probiotics have been investigated for their ability to support the maintenance of oral health through microbiome regulation and protection against pathogens such as *Streptococcus mutans*^8,9^. However, limited research exists regarding the therapeutic use of postbiotics, which refers to the intra- and extracellular components of probiotics.

Previous work by Moman et al.^10^ reported that *Lactobacillus rhamnosus* (GG), *L. reuteri*, and *Streptococcus salivarius* pro- and post-biotics offered significant protection against periodontal pathogens in vitro. Furthermore, the protective effects of GG lysate and supernatant have been observed against *Staphylococcus aureus*, with postbiotic treated cells demonstrating improved cell viability^11^. GG has also been shown to enhance re-epithelialisation in wound healing models^12^. Notably, Prince et al.^13^ observed that live and lysed *L. reuteri* offers protection against *S. aureus* induced cell death in a time dependent manner, with longer probiotic pre-treatment resulting in improved cell viability. However, it was also reported that probiotic treatment post pathogenic infection did not reduce host cell death.

The use of *L. plantarum *to protect against oral pathogens has not been extensively studied, however, its positive influence on oral^14^ and gut^15^ barrier function has been documented. The effects of other *Lactobacilli*, and related non-viable formulations, on gut barrier function have also been thoroughly investigated, with numerous studies reporting increases in tight-junction (TJ) protein expression and enhanced barrier integrity after treatment^16–18 ^as well as modulatory effects in the presence of pathogens^19^. TJ proteins are essential for regulating the passage of substances, including microorganisms, through the epithelial layer^20^. Periodontal pathogens compromise barrier integrity by reducing TJ expression^21^, hindering effective disease management^22^. Therefore, treatments that can increase TJ expression and enhance barrier integrity are of particular interest.

Postbiotics provide a logistical advantage over live cells by overcoming issues associated with viability. The most common way of storing probiotics is freeze drying^23^. This, as well as other formulation solutions, can reduce the viability of the bacterial cells^24^. Cheng et al.^25^ reported that the survival rate of *L. plantarum* after freeze drying was 6.57%, and complex formulations were required to maintain a high level of viability.

In the current study, we have examined the effects of four *Lactobacillus* lysates on oral keratinocytes, observing their prophylactic effects against three periodontal pathogens: *Porphyromonas gingivalis* (PG), *Fusobacterium nucleatum* (FN) *and Aggregatibacter actinomycetemcomitans*(AA)^26^. We studied the ability of *Lactobacillus* lysates to maintain cell viability against intra- and extracellular pathogenic material in addition to live bacteria. Furthermore, we analysed a diverse range of cytokines and assessed the effects of *Lactobacillus* lysates in a wound healing model. Finally, we studied the impact of our lysates on barrier integrity and TJ protein expression.

## Methods

### Cell culture

TR146 cells (buccal carcinoma cell line: ECACC 10032305; obtained from Sigma-Aldrich, Dorset, UK), passage 8–12, were used in all in vitro work. Cells were cultured using T225 culture flasks in Hams F-12 nutrient mix (Sigma-Aldrich, Germany) supplemented with 10% foetal bovine serum (FBS; ThermoFisher Scientific, UK), 1% Penicillin-Streptomycin (Sigma-Aldrich) and 1% L-glutamine (ThermoFisher Scientific). Flasks were incubated at 37 °C and 5% CO_2_ until at least 80% confluency was reached. Trypsin-EDTA (Sigma-Aldrich) was used to detach cells between passages. Cells were frozen in liquid nitrogen vapour in 90% FBS and 10% DMSO (Sigma-Aldrich).

## Bacterial culture, lysate and supernatant preparation

Pathogens: *Porphyromonas gingivalis* ATCC 33277 (PG), *Fusobacterium nucleatum* ATCC 10953 (FN) and *Aggregatibacter actinomycetemcomitans* ATCC 33384 (AA); and probiotics: *Lactobacillus rhamnosus* ATCC 53103 (GG), *Lactobacillus rhamnosus *SD11 (SD)^27,28^, *Lactobacillus reuteri* ATCC 55730 (LR) and *Lactobacillus plantarum* ATCC 10241 (LP), were cultured in Wilkins-Chalgren anaerobe broth (WCB; ThermoFisher Scientific) and incubated in an anaerobic cabinet (Don Whitley, UK) supplied with an anaerobe gas mix (5% CO_2_, 5% H_2_, 90% N_2_), at 37 °C for 48 h.

Cultures were centrifuged at 1520 xg for 10 min to pellet bacterial cells. Supernatants were collected and filtered using a 0.22 μm syringe filter and stored at −20 °C. Bacterial pellets were washed in Dulbecco’s Phosphate Buffered Saline (DPBS; Sigma-Aldrich) and resuspended in 20mL DPBS. The cells were lysed by sonication using a Sonoplus HD4100 Sonicator (BANDELIN electronic GmbH & Co., Germany) on ice and at 100% amplitude, with a pulse-on for 30 s, pulse-off for 10 s; 30 kJ were applied to each sample. Lysates were filtered using a 0.22 μm syringe filter and concentrated using 3 kDa protein concentrators (centrifuged at 3000xg for 90 min at 4 °C). Protein concentration was determined by a Qubit Protein Assay (ThermoFisher Scientific). The relationship between colony forming units (CFU/mL), optical density (OD) and protein concentration of the supernatants and lysates has been included in Supplemental Fig. 1.

Both supernatants and lysates were tested for sterility by plating on Wilkins-Chalgren agar (WCA) and the pH of treatments and media were determined before use.

## Cell culture assays

### Concentration associated effects

TR146 cells were seeded into 48-well cell culture plates (1.2 × 10^5^ cells/mL), using Hams F-12 (Sigma-Aldrich) supplemented with 1% L-glutamine: Hams + L. Cultures were incubated at 37 °C and 5% CO_2 _until 80% confluency was reached. Bacterial lysates or supernatants were added to each well at a range of concentrations (250 µg/mL – 5 µg/mL). For the control (0 µg/mL), treatment was substituted with DPBS for lysates, or DPBS and WCB (1:1) for supernatants. After 24 h, the plates were centrifuged at 200xg for 3 min to pellet dead cells. Cells were then harvested using Accutase (ThermoFisher Scientific) and viability was determined using trypan blue exclusion as previously described^29^.

## Pre-treatment with probiotic lysates

TR146 cells were seeded into 48-well cell culture plates (1.2 × 10^5^ cells/mL), using Hams + L. Cultures were incubated at 37 °C and 5% CO_2_ until 80% confluency was reached. Cells were pre-treated with probiotic lysate (250 µg/mL) for either 0 h (baseline), 2 h, 6 h, 12 h, or 24 h. Pathogen (live, supernatant or lysate) was added to the probiotic-treated cells at the relevant time points for 24 h. Pathogenic lysates and supernatants were added at 250 µg/mL protein concentration. Live bacteria were added at 1 × 10^4^ CFU/mL, as determined by plate counting. The pathogen-only control cells received pre-treatment with DPBS and were challenged with pathogenic material. Control cells were treated and ‘challenged’ with DPBS (lysates and live pathogen) or treated with DPBS and ‘challenged’ with DPBS and WCB 1:1 (supernatants).

After 24 h incubation in the presence of pathogen, the plates were centrifuged at 200xg for 3 min to pellet dead cells. Cells were then harvested using Accutase (ThermoFisher Scientific). Viability was determined using trypan blue exclusion^29^, and the mean viability of the pathogen-only control was deducted from each sample viability value to give the difference from the pathogen control. A diagram depicting the controls used in these experiments can be found in the supplemental material (Supplemental Fig. 2).

### Cytokine analysis

TR146 cells were seeded into 48-well cell culture plates (1.2 × 10^5^ cells/mL) using Hams + L and incubated at 37 °C and 5% CO_2_ until 80% confluency was reached. Cells were treated with probiotic lysates (250 µg/mL) and supernatants were collected at 1.5, 3, 6, 12, and 24 h and stored at −20 °C. Cytokine analysis was performed on the Bio-Plex 200 magnetic bead system (Bio-Rad, UK) using the Human XL Cytokine Panel (BioTechne, R&D Systems).

## Re-epithelialisation

TR146 cells were seeded into 24-well cell culture plates (3 × 10^5^ cells/mL) using Hams + L. Cultures were incubated at 37 °C and 5% CO_2_ until 80–100% confluency was reached. Scratches were made in the centre of the well through the keratinocyte monolayer using a p200 pipette tip. Media was removed and wells were washed twice with DPBS to remove cellular debris. Hams + L, supplemented with 1% FBS, was added along with lysates (250 µg/mL). For the positive control, 1% human keratinocyte growth supplement (HKGS: Thermofisher Scientific) was used in place of treatment, while DPBS was used for the control. Images were then taken at 0 (baseline), 6, 12, 18 and 24 h at 5x magnification (Leica, Germany). Scratch size was measured using Image-Pro version 10 (Media Cybernetics Inc, Rockville, MD).

## Trans-epithelial electrical resistance (TEER)

TR146 cells were seeded at a density of 2 × 10^5^ cells/mL into 12 mm, 0.4 μm pore transwells using Hams + L and incubated at 37 °C and 5% CO_2_ until 80% confluency was reached. Media was removed and replaced with EP + S: EpiLife medium containing 60 µM calcium (ThermoFisher Scientific), supplemented with an additional 1.44mM calcium (ThermoFisher Scientific) and 1% HKGS. Cultures were incubated for 24 h before TEER measurements (day 1) were taken using a World Precision Instruments Evometer fitted with Chopstick electrodes (WPI, UK). Apical media was removed and replaced with 400µL EP + S and 100µL of probiotic lysate or a combination of 50µL of probiotic lysate and 50µL of PG supernatant (final apical protein concentrations 100 µg/mL). Following a further 24 h incubation, TEER measurements were repeated (day 2). Cultures were then maintained for a further 12 days with TEER measurements as well as apical media and treatment changes, performed every 2–3 days. Basal media changes were performed every 4–6 days.

### Claudin-1 expression

TR146 cells were seeded into 24-well cell culture plates (2 × 10^5^ cells/mL) using Hams + L and incubated at 37 °C and 5% CO_2_ until 80% confluency was reached. Cells were treated with pathogenic or probiotic lysates (250 µg/mL) for 24 h. RIPA buffer (Thermofisher Scientific) was subsequently used to lyse cells and preserve protein. Protein concentration was determined using a Bradford assay and normalised for Western blot.

Samples containing 40 µg of protein were loaded into 4–12% agarose precast gels (Thermofisher Scientific) and run at 100 V for 75 min. Proteins were transferred to a nitrocellulose membrane using the trans-blot turbo blotting system (Bio-Rad). The membrane was blocked with skimmed milk (1 h) before being incubated with a primary claudin-1 or β-actin antibody (Thermofisher Scientific or abcam respectively) for 1 h. An HRP-conjugated secondary antibody (Biolegend, UK) was subsequently applied for 45 min, followed by the addition of a chemiluminescent reagent for 1 min. Wash steps were performed in between incubations using 1x Tween 20. Protein bands were visualized using a chemiluminescent reader (Azure Biosystems, US) and relative abundance of claudin-1 was determined by calculating the band intensity (ImageJ software), determining the ratio to the control, and normalizing to β-actin expression.

### Statistical analyses

All statistical analyses were performed using GraphPad Prism 10.0.2 (GraphPad Software, US). Two-way ANOVA with a Fishers LSD test was used for viability and re-epithelialisation data. After determining normal distribution, Two-way ANOVA with a Dunnett’s posthoc test was used for statistical analysis of the cytokine dataset. One-way ANOVA with a Fishers LSD test was used for statistical analysis of the TEER data and a two-tailed unpaired t-test was used to calculate statistical significance of the western blot data. For cytokine data, in-text p-values have been summarized; all significant p-values are shown in full in Supplemental Table 1.

## Results

### Concentration associated effects

**Fig. 1:**
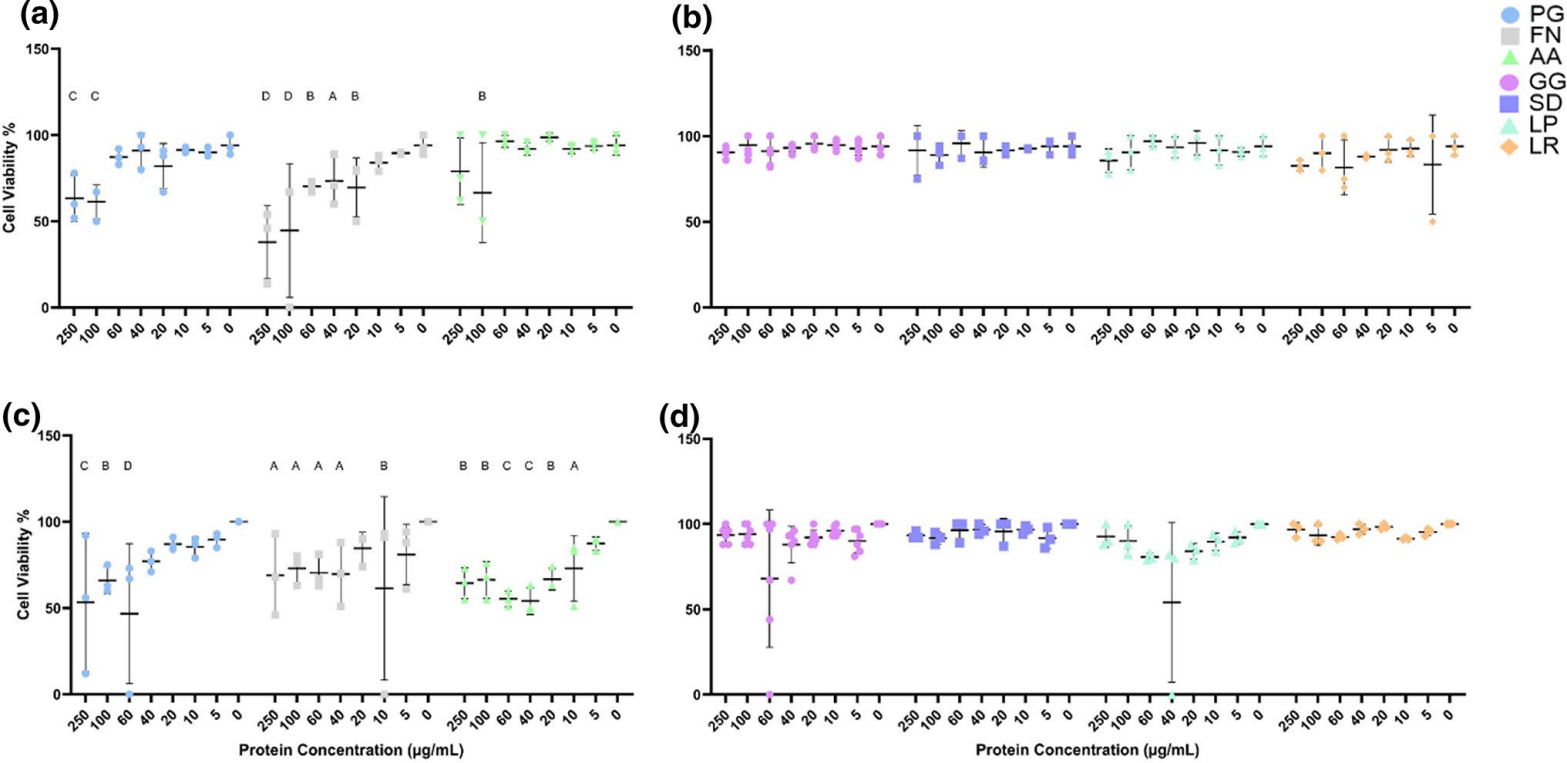
Viability of TR146 cells treated with varying concentrations (250 µg/mL – 5 µg/mL) of pathogenic (**a** &** b**) or probiotic (**c** &** d**) lysates (**a** &** b**) or supernatants (**c** &** d**). *Porphyromonas gingivalis* (PG), *Fusobacterium nucleatum* (FN), *Aggregatibacter actinomycetemcomitans* (AA), *Lactobacillus rhamnosus* (SD), *Lactobacillus plantarum* (LP), *Lactobacillus reuteri* (LR) *n* = 3. *Lactobacillus rhamnosus* (GG) *n* = 6. Control (0 µg/mL) represents TR146 cells where DPBS was added in place of treatment (Lysates) or DPBS and WCB (supernatants). Bars represent the mean+/- SD. Two-way ANOVA A=(*p* < 0.05), B=(*p* < 0.01), C=(*p* < 0.001), D=(*p* < 0.0001).

To observe the toxicity of postbiotics and to determine a lethal pathogen concentration, TR146 cells were treated with various concentrations (250 µg/mL – 5 µg/mL) of lysates and supernatants. Cell viability after 24 h treatment with bacterial lysate or supernatant is shown in Fig. 1. Increased pathogenic lysate concentration is strongly associated with decreased cell viability. When the control is compared to treatment with 250 µg/mL, PG and FN significantly reduce viability (*p* = 0.0003 and *p* < 0.0001 respectively), whereas AA demonstrates trends towards reduced viability (*p* = 0.0708). Conversely, no relationship was found between increased probiotic lysate concentration and cell viability (*p* > 0.05). Similarly to the lysates, the pathogens display a significant relationship between increased supernatant concentration and decreased cell viability. At the highest concentration of treatment, PG, FN and AA significantly reduce cell viability (*p* = 0.0002, *p* = 0.0135 and *p* = 0.0046). No difference was found between increased probiotic supernatant concentration and decreased viability (*p >* 0.05) and are therefore non-toxic at high concentrations.

This data demonstrates the toxic effects of pathogenic material at 250 µg/mL and therefore this concentration was selected as a significant challenge to the keratinocytes. Furthermore, postbiotics at this concentration demonstrated non-toxic effects.

### Pre-treatment analysis: probiotic lysate vs. pathogenic lysate

**Fig. 2:**
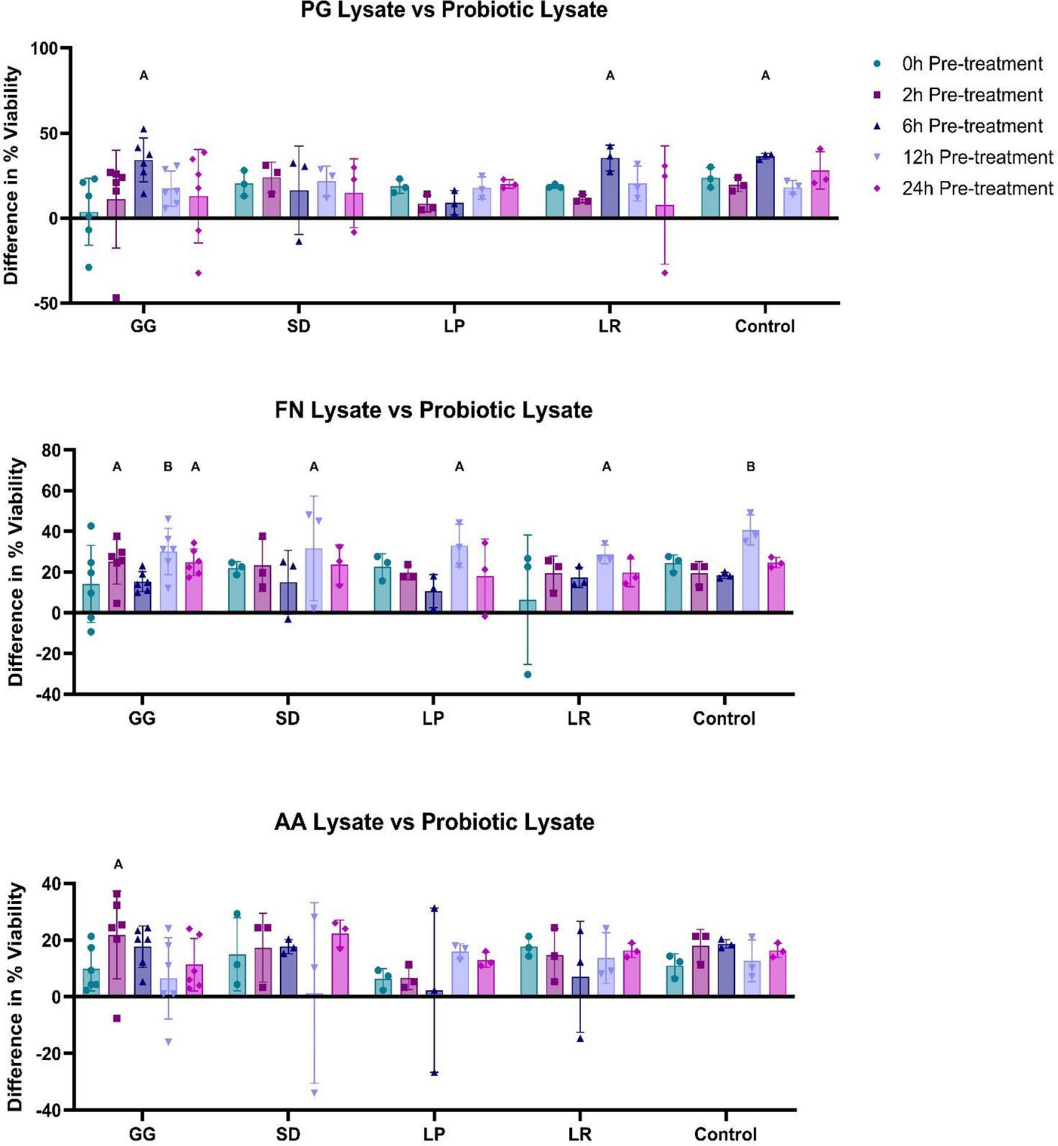
Differences in cell viability compared to the pathogen only control (sample cell viability minus the mean viability of the pathogen-only control). TR146 cells were pre-treated with probiotic lysates (250 µg/mL) for 0, 2, 6, 12 and 24 h. Pathogenic lysates (250 µg/mL) were then added for 24 h after pre-treatment. *Porphyromonas gingivalis* (PG), *Fusobacterium nucleatum* (FN), *Aggregatibacter actinomycetemcomitans* (AA), *Lactobacillus rhamnosus* (SD), *Lactobacillus plantarum* (LP), *Lactobacillus reuteri* (LR) *n* = 3. *Lactobacillus rhamnosus* (GG) *n* = 6. Control represents cells which were treated with DPBS and were not challenged with pathogenic lysate. Bars represent the mean+/- SD. Statistics are compared to the pathogen-only control: Two-way ANOVA: A=(*p* < 0.05), B=(*p* < 0.01), C=(*p* < 0.001), D=(*p* < 0.0001).

Intracellular components of pathogens are toxic to cells (Fig. 1), therefore, to assess whether probiotic lysates could protect against intracellular pathogenic material, and if longer pre-treatment enhanced protection, TR146 cells were treated with probiotic lysates for 0, 2, 6, 12, and 24 h and challenged with pathogenic lysates for 24 h; results are shown as difference in percent viability to the pathogen-only control (Fig. 2). On average, all probiotic treatment conditions (60/60) led to an observed improvement in cell viability compared to the pathogen only control, 15% (9/60) of which were statistically significant (*p* < 0.05). No significant difference was found between pre-treatment timepoints or between individual probiotic performance (*p* > 0.05).

The average effects of 24 h pre-treatment with probiotics is shown in Supplemental Fig. 3A. Cell viability was improved against FN by 22.2% (*p* = 0.0113), with non-significant trends towards improved cell viability being observed against PG (13.6%, *p* = 0.1227) and AA (14.87%, *p* = 0.0845).

### Pre-treatment analysis: probiotic lysate vs. pathogenic supernatant

**Fig. 3:**
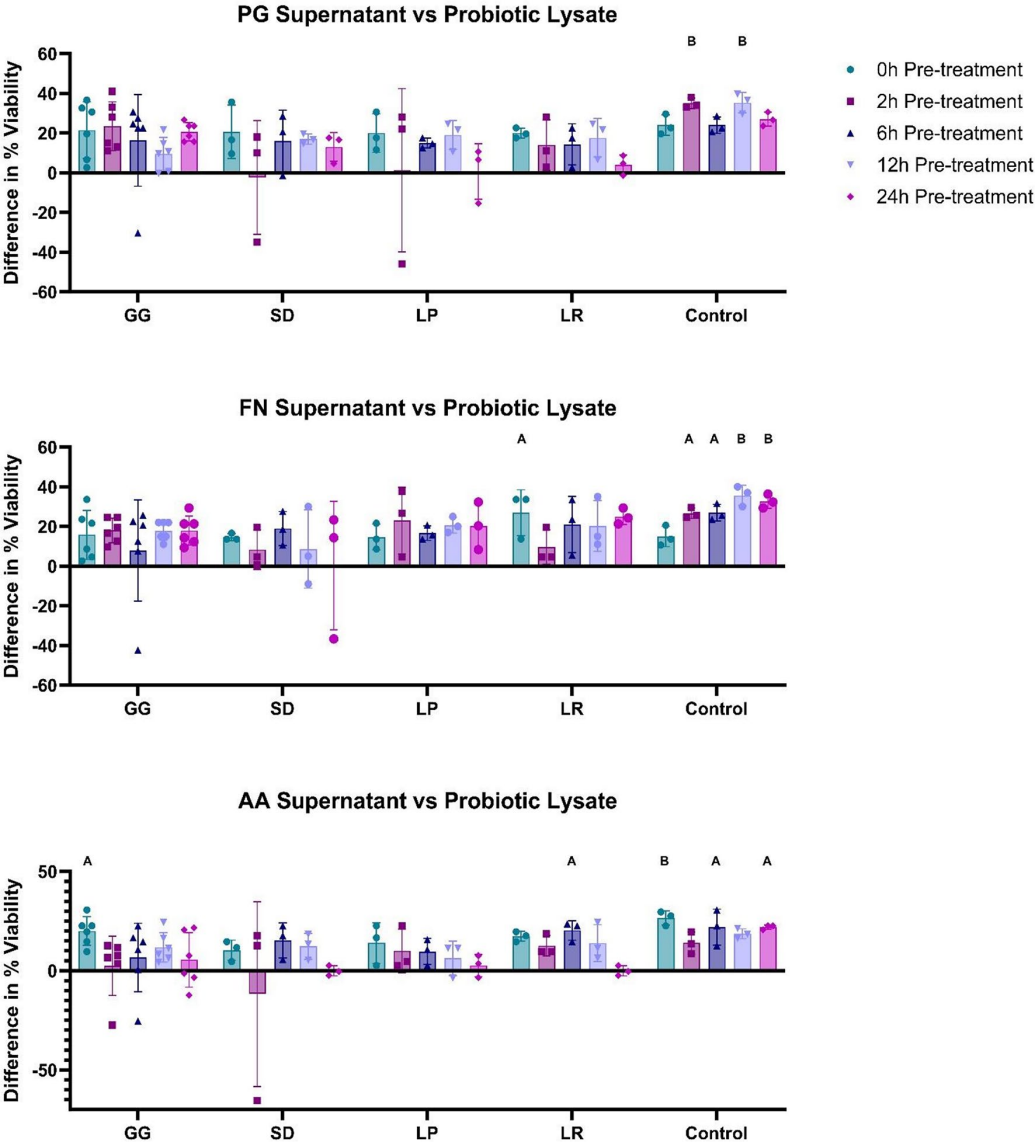
Differences in cell viability compared to the pathogen only control (sample cell viability minus the mean viability of the pathogen-only control). TR146 cells were pre-treated with probiotic lysates (250 µg/mL) for 0, 2, 6, 12 and 24 h. Pathogenic supernatants (250 µg/mL) were then added for 24 h after pre-treatment. *Porphyromonas gingivalis* (PG), *Fusobacterium nucleatum* (FN), *Aggregatibacter actinomycetemcomitans* (AA), *Lactobacillus rhamnosus* (SD), *Lactobacillus plantarum* (LP), *Lactobacillus reuteri* (LR) *n* = 3. *Lactobacillus rhamnosus* (GG) *n* = 6. Control represents cells which were treated with DPBS and WCB and were not challenged with pathogenic supernatant. Bars represent the mean+/- SD. Statistics are compared to the pathogen-only control: Two-way ANOVA: A=(*p* < 0.05), B=(*p* < 0.01), C=(*p* < 0.001), D=(*p* < 0.0001).

As shown in Fig. 1, the secreted components of pathogens can be toxic to cells. To determine the prophylactic effects of probiotic lysates against secreted pathogenic material, TR146 cells were treated with probiotic lysates for 0, 2, 6, 12, and 24 h and subsequently challenged with pathogenic supernatants for 24 h (Fig. 3). Results are shown as difference in cell viability compared to the pathogen only control.

In almost all cases (54/60), treatment with probiotic lysates lead to an observed improvement in cell viability, 5% of which (3/60) were found to be significant (*p* < 0.05). No significant difference was found between pre-treatment timepoints or between probiotics (*p* > 0.05). As shown in Supplemental Fig. 3B, after 24 h pre-treatment, probiotic lysates significantly improved cell viability against FN (16.3%: *p* = 0.0251). Furthermore, trends towards improved cell viability against PG was shown by an 11.8% improvement in viability compared to the positive control (*p* = 0.1019).

### Pre-treatment analysis: probiotic lysate vs. live pathogen

**Fig. 4:**
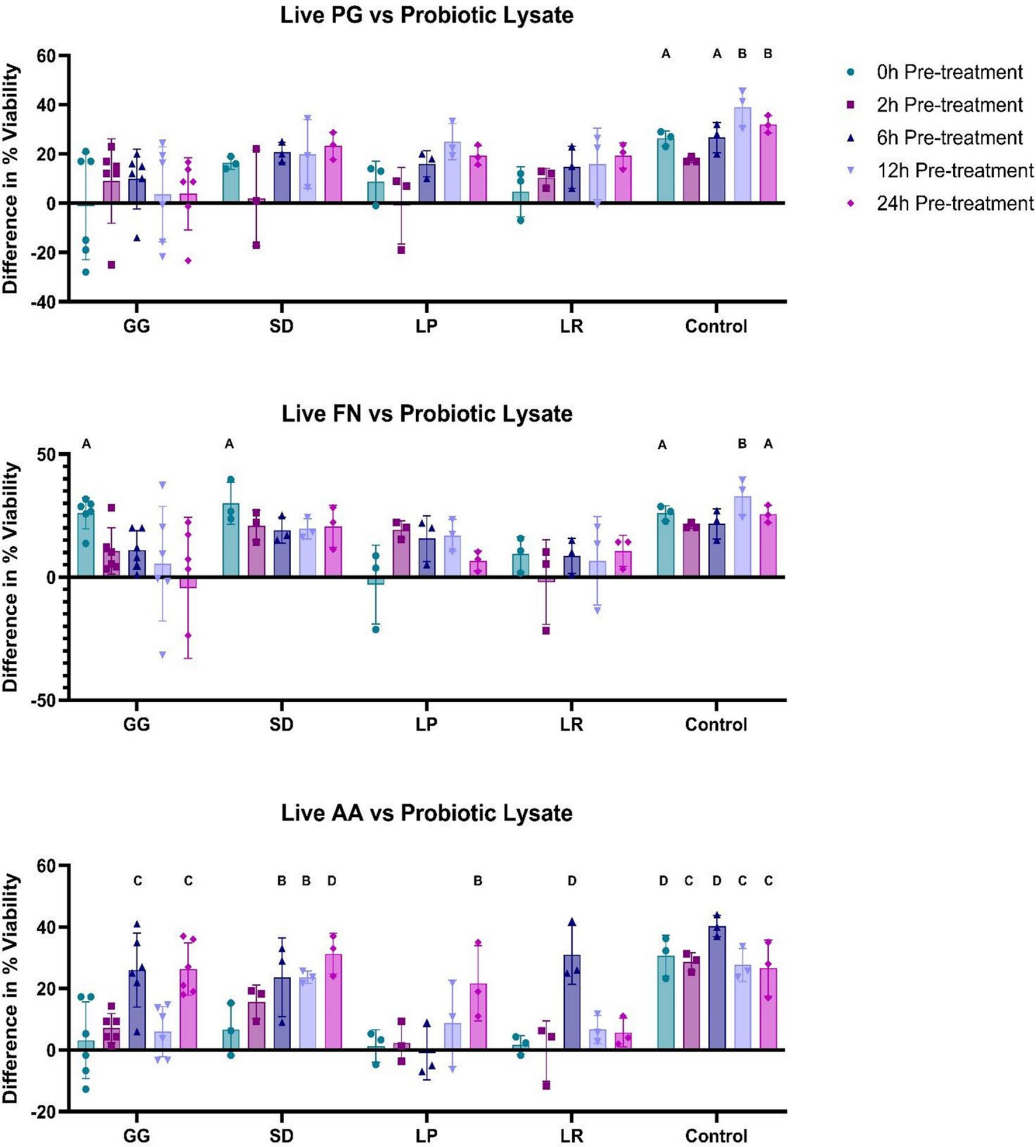
Differences in cell viability compared to the pathogen only control (sample cell viability minus the mean viability of the pathogen-only control). TR146 cells were pre-treated with probiotic lysates (250 µg/mL) for 0, 2, 6, 12 and 24 h. Live pathogen (10^4^) was then added for 24 h after pre-treatment. *Porphyromonas gingivalis* (PG), *Fusobacterium nucleatum* (FN), *Aggregatibacter actinomycetemcomitans* (AA), *Lactobacillus rhamnosus* (SD), *Lactobacillus plantarum* (LP), *Lactobacillus reuteri* (LR) *n* = 3. *Lactobacillus rhamnosus* (GG) *n* = 6. Control represents cells which were treated with DPBS and were not challenged with pathogen. Bars represent the mean+/- SD. Statistics are compared to the pathogen-only control: Two-way ANOVA: A=(*p* < 0.05), B=(*p* < 0.01), C=(*p* < 0.001), D=(*p* < 0.0001).

After observing the prophylactic effects of *Lactobacillus* lysates against non-viable pathogenic material we aimed to simulate a live pathogenic infection. TR146 cells were treated with probiotic lysates for 0, 2, 6, 12, and 24 h followed by a 24 h challenge with live pathogen (10^4^ CFU/mL) (Fig. 4). Results are shown as difference to the pathogen only control. As before, almost all treatments led to an observed improvement in viability compared to the pathogen-only control (54/60), none of which were significant for PG (0/20) and two of which were significant against FN (2/20). However, against AA, 35% (7/20) of treatments significantly improved cell viability compared to the pathogenic control (*p* < 0.01). No correlation was found between longer pre-treatment and improved viability against PG or FN (*p* > 0.05), whereas against live AA, longer pre-treatment led to improved cell viability, with GG, SD, and LP significantly improving cell viability after 24 h pre-treatment (*p* < 0.001).

On average, pre-treatment with postbiotics for 24 h also showed trends towards improved viability against PG (13.9%, *p* = 0.1162) but limited effects against FN (*p* = 0.5043) (Supplemental Fig. 3C).

### Cytokine analysis

**Fig. 5:**
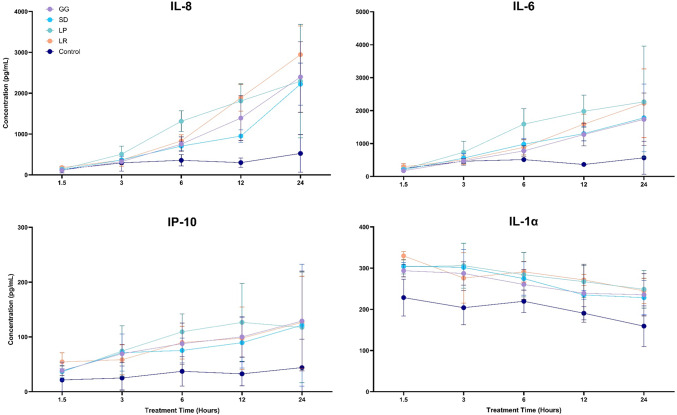
Cytokine expression of TR146 cells treated with probiotic lysates (250 µg/mL) for 1.5, 3, 6, 12 and 24 h. *Lactobacillus rhamnosus* (GG) *n* = 12, *Lactobacillus rhamnosus* (SD), *Lactobacillus plantarum* (LP), *Lactobacillus reuteri* (LR) *n* = 6. Control refers to cells where DPBS were added in place of treatment (*n* = 4). Bars represent the mean+/- SD.

The impact of postbiotics on keratinocyte cytokine expression was assessed to determine any time-dependent effects. Figure 5 (IL-8, IL-6, IP-10, IL-1α) and Supplemental Fig. 4 (TNF-α, IL-10, IL-1β, TGF-β) show results for eight cytokines after treatment with 250 µg/mL of probiotic lysates for 1.5, 3, 6, 12 and 24 h. In-text p-values have been summarized, individual values are shown in Supplemental Table 1. Increases in IL-8, IL-6 and TNF-α concentration were recorded after 6 h of postbiotic treatment and became significant after 12 h (*p* < 0.05); all probiotics showed a significant increase in these cytokines after 24 h of treatment (*p* < 0.05). IP-10 concentration also increased with time however significance was only recorded for GG and LR after 24 h (*p* < 0.05). For all probiotics, IL-1β concentration increased significantly after 1.5 h pre-treatment (*p* < 0.05) and decreased over time to levels like the control by 24 h. Similarly, IL-1α increased significantly after 1.5 h pre-treatment (*p* < 0.05) for all probiotics however it maintained levels higher than the control for 24 h (*p* < 0.05). Finally, TGF-β concentration significantly increased 3 h after treatment with all probiotics (*p* < 0.05) and decreased over time to levels like the control after 24 h.

Between probiotics, some significant differences were recorded. Generally, treatment with LR led to the greatest cytokine response, demonstrating significantly higher IL-8, TGF-β, IL-6, IL-10 and TNF-α expression than SD and GG at various timepoints (*p* < 0.05). LP also demonstrated an increase in IL-8 compared with SD after 12 h of treatment (*p* < 0.01), as well as an increase in IL-6 compared to GG after 6 h of treatment (*p* < 0.05).

IFN-γ and IL-17 A were also considered in this panel however neither were produced at a high enough concentration to be detected.

### Re-epithelialisation

**Fig. 6:**
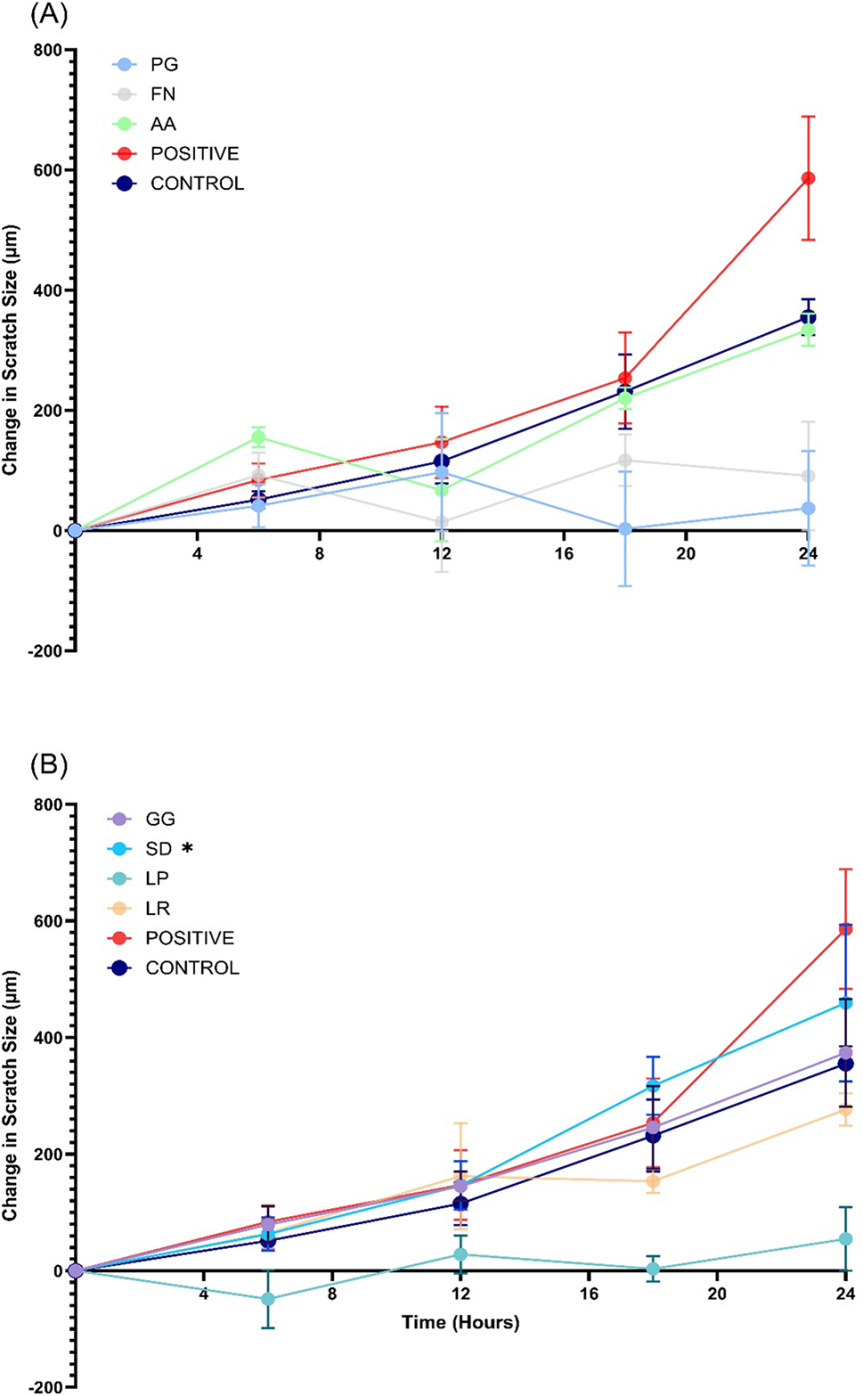
Difference in scratch size compared to 0 h (baseline) after treatment with lysates (250 µg/mL). Pathogens (**A**) *Porphyromonas gingivalis* (PG), *Fusobacterium nucleatum* (FN), *Aggregatibacter actinomycetemcomitans* (AA), and probiotics (**B**) *Lactobacillus rhamnosus* (SD), *Lactobacillus plantarum* (LP), *Lactobacillus reuteri* (LR) *n* = 3, *Lactobacillus rhamnosus* (GG) *n* = 6. Control represents cells treated with DPBS in place of lysate. Positive represents cells treated with 1% HKGS. Bars represent the mean+/- SD. Two-way ANOVA *(*p* < 0.05) relative to the control at 24 h.

Pathogen associated damage can compromise epithelial barrier integrity. To determine if postbiotics can improve healing, re-epithelialisation of TR146 cells after exposure to pathogenic and probiotic lysates was observed (Fig. 6). Representative scratch images are shown in Supplemental Fig. 5. When compared to the negative control, treatment with SD significantly improved re-epithelialisation at 24 h (*p* = 0.0229), with trends towards improvements at earlier timepoints (*p* = 0.0606, 18 h). This suggests SD lysates could have beneficial properties in restoring compromised barrier integrity. GG and LR had no significant impact on re-epithelialisation and demonstrated similar results to the control (*p* > 0.05). Interestingly, LP significantly inhibited re-epithelialisation (*p* < 0.0001) and showed comparable results to pathogens FN and PG.

### Trans-epithelial Electrical Resistance (TEER)

**Fig. 7:**
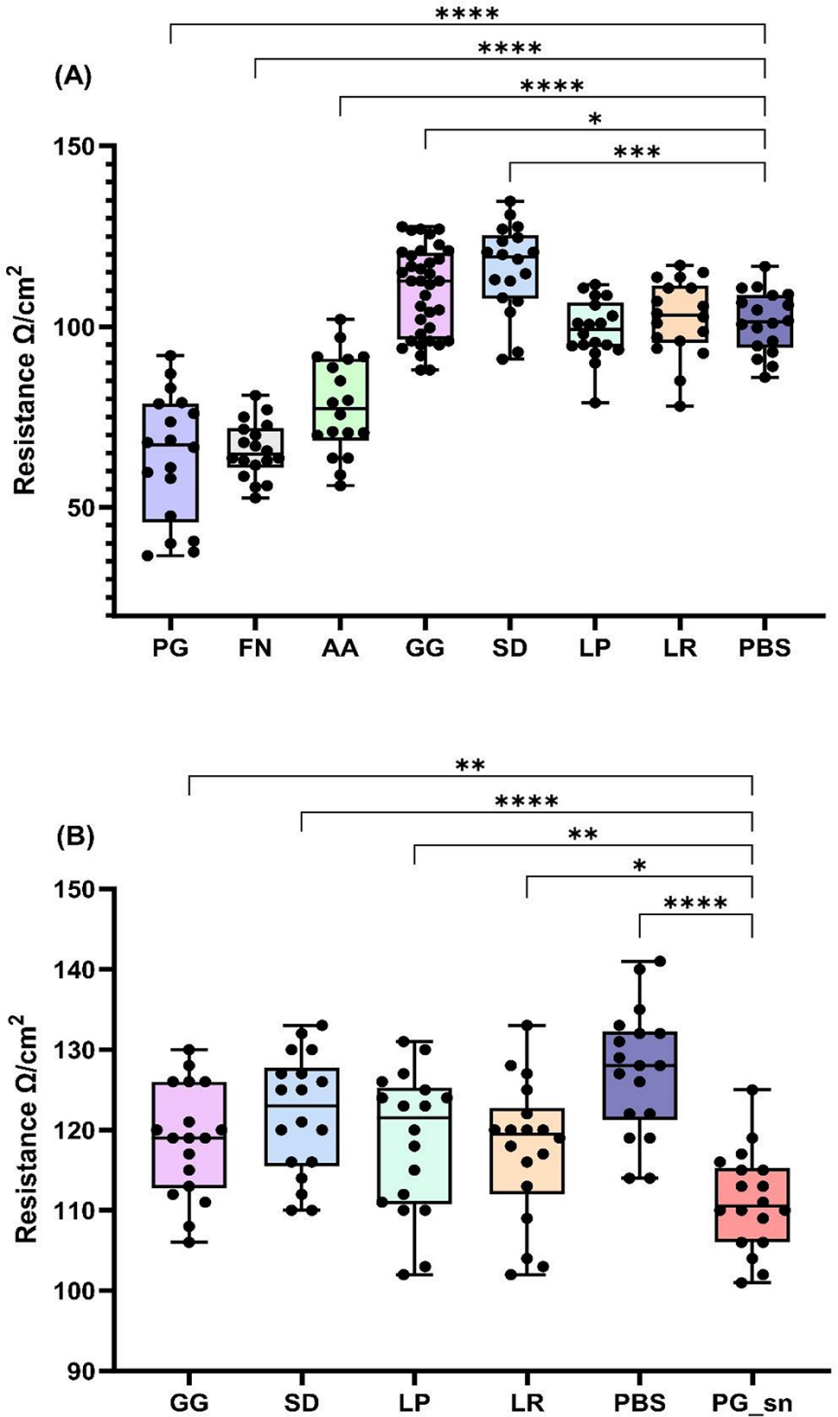
Trans-epithelial electrical resistance (TEER). TR146 cells treated with 100 µg/mL of pathogenic or probiotic lysate (**A**) or a combination of 50 µg/mL of probiotic lysate and 50 µg/mL PG supernatant (**B**). PBS represents cells where treatment was replaced by DPBS. PG_sn (**B**) represents cells treated with 50 µg/mL PG supernatant and DPBS in place of probiotics. Porphyromonas gingivalis (PG), Fusobacterium nucleatum (FN), Aggregibacter actinomycetemcomitans (AA), Lactobacillus rhamnosus (SD), Lactobacillus plantarum (LP), Lactobacillus reuteri (LR). (*n* = 3) Lactobacillus rhamnosus (GG) *n* = 6 (**A**) *n* = 3 (**B**). Time-course data from after treatment has been added to boxplots in a non-time dependent manner. One-way ANOVA *(*p* < 0.05), **(*p* < 0.01), ***(*p* < 0.001), ****(*p* < 0.0001).

The maintenance of epidermal barrier integrity is essential for the management of periodontal pathogens. TEER was used to determine if probiotic lysates could enhance or maintain the epithelial barrier (Fig. 7: A) or moderate the effects subsequent exposure to PG supernatant (Fig. 7: B). Results obtained after treatments have been combined into boxplots in a non-time dependent manner.

On average, over the 12-days of treatment, GG and SD significantly increased TEER by 11.3 Ω/cm^2^ (*p* = 0.0165) and 18.0 Ω/cm^2^ (*p* = 0.0003) respectively. Probiotics LR and LP showed a comparable TEER to the control, suggesting they have minimal effect on the expression of TJ proteins (*p* > 0.05). Pathogenic lysates significantly reduced TEER compared to the control (*p* < 0.0001), however TEER of cells exposed to AA began to increase after day seven.

Addition of PG supernatant without probiotic treatment led to a significant reduction in TEER (17.5 Ω/cm^2^: *p* < 0.0001). However, in the presence of all probiotics, TEER was significantly increased when compared to the pathogen-only control, demonstrating a prophylactic effect on barrier integrity. SD conveyed the best protection (12.5 Ω/cm^2^ increase in TEER: *p* < 0.0001) followed by LP (11 Ω/cm^2^ increase: *p* = 0.0053), LR (9 Ω/cm^2^ increase: *p* = 0.0155) and GG (8.5 Ω/cm^2^ increase: *p* = 0.0047).

### Claudin-1 expression

**Fig. 8:**
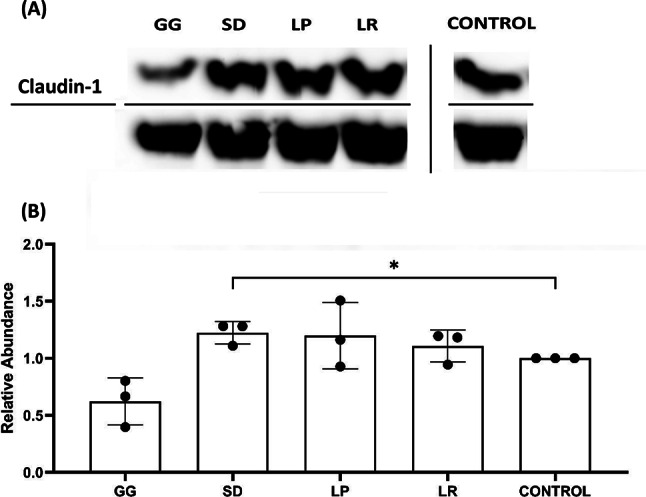
(**A**) Representative western blot. TR146 cells treated with 250 µg/mL of probiotic lysate. Control represents cells where treatment was replaced by DPBS. Lactobacillus rhamnosus (GG), Lactobacillus rhamnosus (SD), Lactobacillus plantarum (LP), Lactobacillus reuteri (LR). (**B**) Relative abundance of claudin-1 quantified by western blot as in A (*n* = 3). Bars represent mean+/- SD. Unpaired t-test *(*p* < 0.05).

To determine if increased TEER correlated with claudin-1 expression, western blot analysis was performed (Fig. 8). Treatment with SD led to a 22% increase in claudin-1 (*p* = 0.0174), with LP and LR demonstrating an observed increase in claudin-1 by 20% (*p* = 0.1513) and 11% (*p* = 0.1283) respectively. Interestingly, treatment with GG significantly reduced claudin-1 concentration (*p* = 0.0337) suggesting increases in TEER were mediated by other factors.

## Discussion

We have determined a high concentration at which pathogens significantly reduce cell viability, and postbiotics are non-toxic. Many studies do not consider protein concentration in lysate preparation^7,30,31^ despite numerous investigations demonstrating that proteins from *Lactobacillus *species, particularly membrane components, can have positive effects on host cells^32–34^. Our data indicates that increased protein concentration can lead to greater changes in cell viability and have demonstrated a significant relationship between the protein concentration of a lysate and its effects on oral epithelial cells. Additionally, this study presents data linking CFU/mL and OD, to intra- and extra-cellular protein concentration (Supplemental Fig. 1). Interestingly, we observed that AA and LR had the lowest protein concentrations in their lysates but high levels in their supernatants. This was especially notable for AA, which showed strong extracellular protein expression despite having a low CFU/mL count, demonstrating its tendency to form biofilms^35^. Additionally, probiotics GG and LP exhibited the highest lysate protein concentrations; however, while GG showed low supernatant protein levels, LP had one of the highest.

Our study utilised trypan blue staining to assess various effects on cell viability. This is a commonly used method, particularly suited to co-culture models where metabolic assays like MTT can be unsuitable due to interference from microbial metabolism. However, it is important to note that trypan blue staining may not detect cells with impaired or failing metabolism that retain an intact membrane, potentially overlooking a specific subset of non-viable cells^36,37^.

Orally-relevant protective effects of probiotics have been reported in numerous studies using live formulations. Morales et al.^38^ for example, reported that *L. rhamnosus* can control chronic periodontitis in a clinical setting. Similarly, Ince et al.^39^ reported modulatory effects in volunteers with chronic periodontitis when administered lozenges containing *L. reuteri*. This has also been observed in some studies in the context of postbiotics^10,11,13^ however, to our knowledge previous investigations have not considered the pathogens included in this study.

Significant prophylactic effects were seen against live AA after 24 h pre-treatment with GG, SD and LP; LR also demonstrated a non-significant protective effect. AA can cause aggressive periodontitis and bone resorption^35^; it has been detected in 90% of localised periodontitis infections and is therefore characterised as one of the leading causes of periodontitis^40^. Future studies could consider why keratinocytes are protected from AA in the presence of *Lactobacillus* lysates.

*Lactobacilli* are known to alter cytokine production in host cells by the expression of peptidoglycans in the cell wall. Binding of *Lactobacilli *peptidoglycans to toll-like receptors (which are highly expressed on keratinocytes) activates the transcription factor NF-κB^41^, which has a role in regulating cytokine production, as well as genes relating to differentiation and proliferation^42^. In our study, longer pre-treatment led to greater increases in pro-inflammatory cytokines. The release of inflammatory molecules in response to *Lactobacillus* strains has previously been reported. For example, Mohammedsaeed et al.^11^, reported that the expression of 14 molecules relating to the inflammatory response were increased by GG lysate, with TNF-α and IP-10 both being upregulated. In addition, Lee et al.^43^, showed that live *L. rhamnosus* increased TNF-α and IFN-γ expression in skin keratinocytes, while Jiang et al.^44^ that live *L. acidophilus* significantly upregulated the expression of IL-1α and IL-1β in Caco-2 cells.

Despite the association of periodontitis with inflammation, the release of inflammatory cytokines in response to postbiotics could be a key element of their protective effects. TNF-α is associated with antimicrobial host response; recruiting immune cells and increasing vascular permeability to allow access of immune cells^45^. IL-6 has been associated with aggressive periodontitis and inflammation, with high concentrations being found in the saliva of those affected with periodontal disease^46^, but it is also a potent stimulator of cell differentiation, boosting keratinocyte growth and survival, as well as enhancing the immune response^47^. Irwin and Myrillas^48 ^suggest IL-6 may play both an immunopathogenic pathogenic and protective role in oral disease. Similarly, higher levels of IL-8 are often found in infected periodontal tissue and are associated with periodontitis^49^. Despite this, IL-8 is also an essential part of the immune response and a key element in fighting infection^50^.

IL-1α and IL-1β concentration increased significantly 1.5 h after treatment (although IL-1β secretion was relatively low). While both cytokines are associated with severe periodontitis due to their inflammatory nature and promotion of bone reabsorption, this appears to be a time-dependent effect. It has been noted that low-level increases of IL-1 can be beneficial to host health and lack of IL-1 secretion in response to infection can result in a reduced ability to control infection^51^. It is therefore important to quantify changes demonstrated in the presence of probiotics in this study. Sanchez et al.^52^ demonstrated that salivary IL-1β concentration in volunteers with moderate periodontitis was 500% higher than healthy participants, this is a far more considerable increase than shown in our in vitro study. Furthermore, Kim et al.^53^ showed the mean salivary levels of IL-1α in those with periodontal disease was 590.78 pg/mL, and healthy volunteers were 343.60 pg/mL. As before, those with periodontal disease demonstrate a much higher IL-1α concentration than shown above. Therefore, although levels of the IL-1 family show an increase, this could be an essential mechanism of action of postbiotics.

TGF-β decreased over time for all samples including the control, although expression was relatively low. For all probiotics, expression was significantly higher when compared to the control, and the time it took for these levels to decrease was maintained for longer. TGF-β plays a key role in the management of periodontitis as a powerful stimulator of tissue regeneration and barrier enhancement^54^ therefore, significant increases demonstrated by our probiotic lysates could be an essential aspect in the management of periodontitis.

IP-10 demonstrated a time-dependent increase, with longer pre-treatment resulting in higher expression. IP-10 is a chemoattractant essential for recruiting immune cells to infected tissue^55^. Gemmell et al.^56^ found that IP-10 levels decreased with increasing inflammation in periodontal disease. However, it has been noted that IP-10 concentration generally increases in infected periodontal tissue^55^. Notably, high levels of IP-10 has been shown to inhibit wound healing in mice^57^.

IL-10 levels remained comparable to the control for most probiotics, with non-significant increases shown for LR. IL-10 is an important regulatory cytokine, and can reduce inflammation in periodontal disease; however, it has been suggested that this can lead to a lack of immune cell recruitment contributing to the persistence of bacterial presence in infected tissue^58^.

Overall, the cytokine data presented in this document and the wider literature is complex. Studies have reported conflicting arguments for almost all cytokines, for example, Kim et al.^53^ present no significant difference in salivary TNF-α and IL-8 between individuals with and without periodontitis. Furthermore, as discussed for the IL-1 family, the degree of concentration change is relevant. Increased inflammatory cytokine production results in immune cell recruitment, it is only when cytokines are persistently overproduced for long periods that their effects can become immunopathogenic. Moreover, Zhang et al.^45^ found a relationship between the short (14 days) and long term (35 days) effects of TNFα and IL-1 secretion, noting that blocking the activity of these cytokines in non-human primates with lab-induced periodontitis, improved management of the disease in the short term, but led to disease progression over the long term. The results discussed in this document do not consider cytokine release for an extended treatment period. Finally, despite the increases in inflammatory markers following treatment with postbiotics, the cell viability data presented in this study demonstrates the protective effects of postbiotic lysates, suggesting that alterations to cytokine secretion do not cause any adverse effect and may contribute to the mechanism of action ‘priming’ the immune system to facilitate bacterial clearance.

The ability for *L. rhamnosus* to improve re-epithelialisation has been previously reported. Mohammedsaeed et al.^12^ found re-epithelialisation of epidermal keratinocytes was significantly enhanced in the presence of *L. rhamnosus* lysates. Given the association of periodontitis with barrier breakdown^59^, an important characteristic of a periodontitis treatment could be regulating barrier function. Probiotic lysates may contribute to improved wound healing by upregulating cytokines such as IL-1α, IL-1β, TGF- β, IL-6, and TNF-α, all known to enhance wound healing, cell migration, and proliferation^60^. Notably, the ability of *L. rhamnosus *lysates to accelerate re-epithelialisation, highlights the potential of postbiotics in facilitating immediate wound healing and closure. Interestingly, LP significantly inhibited wound closure when compared to the control, contradicting previous findings^61,62^ and warranting further investigation.

Increases in TEER after treatment with *Lactobacillus *probiotics have been previously reported in gut models^15,63^. Sultana et al.^14^ demonstrated that GG lysate significantly increases TEER of human keratinocytes, with higher doses (10^8 ^CFU/mL) being increasingly effective, which the authors suggest could be facilitated by interaction with toll-like receptors on the keratinocyte surface. Given that disruption of the epithelial barrier and intrusion by periodontal pathogens leads to inflammation and chronic infection^59^, improving barrier integrity could be one way in which *Lactobacillus *lysates exert their prophylactic effects^64^.

In contrast to our findings, claudin-1 expression has been shown to increase after exposure to *L. rhamnosus *lysate^14^. Furthermore, live *L. plantarum* can increased the expression of claudin-1, as well as occludin and ZO-1 in the guts of IL-10 knockout mice^65^. Yang et al.^63^ has previously explored the effects of *L. reuteri *on tight-junction expression pig intestine models, demonstrating that LR can increase claudin-1 expression even in a lipopolysaccharide challenged environment. Claudin-1 is a major component of keratinocyte TJ’s, with downregulation in the skin barrier being linked water-loss conditions such as dermatitis^66^. It has also been shown that lipopolysaccharides can reduce TJ expression^63 ^suggesting that periodontal pathogens may be able to supress TJ’s^67^ leading to a compromised barrier. Decreases in claudin expression after exposure to GG has not been previously reported. We suggest the increase in TEER could be driven by another member of the TJ family that has not been investigated here; occludin and ZO-1 have all been shown to increase after treatment with GG^14^.

The mechanism by which *Lactobacillus *probiotics enhance the epithelial barrier is not fully understood. However, changes in cytokine expression could be one mechanism of action. IL-6 (which significantly increased for all postbiotics) has been shown to have protective effects on epithelial barriers in gut models^68^. Furthermore, Stolte et al.^69^ demonstrated that, although IL-1β often causes barrier compromise in intestinal models, in oral cells its presence significantly increases IL-6 and IL-8 expression, leading to an enhancement in TEER and claudin-1. These findings validate that our lysates could increase TEER by raising IL-6, IL-8 and IL-1β expression.

## Conclusion

*Lactobacillus* lysates exhibited prophylactic effects against high concentrations of intra and extracellular pathogenic material as well as live pathogens. Moreover, treatment with postbiotics significantly altered cytokine expression of TR146 cells. Despite increases in some inflammatory cytokines, the viability, re-epithelialisation, and TJ data demonstrated positive effects after exposure to *Lactobacillus* lysates. Furthermore, increases in inflammatory cytokines are essential for immune recruitment, access to infected tissue, keratinocyte proliferation and barrier maintenance. We suggest a key mechanism of action of probiotic lysates could be the ability to ‘prime’ the host’s immune system. Furthermore, the fact that probiotic lysates maintain and improve barrier integrity could enhance disease management, representing a potential periodontitis therapeutic. Future work could confirm the mechanisms by which *Lactobacillus* lysates are able to protect oral keratinocytes from periodontal pathogens. Characterisation and isolation of key proteins within the lysates would be of particular of interest, to examine their effects individually.

## Electronic supplementary material

Below is the link to the electronic supplementary material.

Supplementary Material 1

Supplementary Material 2

Supplementary Material 3

Supplementary Material 4

## Data Availability

Data available upon request of the corresponding author.
