# Supplementary material for: Lactobacillus lysates protect oral epithelial cells from pathogen-associated damage, increase secretion of pro-inflammatory cytokines and enhance barrier integrity
Source: Sci Rep. 2025 Feb 18;15:5894. doi: 10.1038/s41598-025-86914-y (PMC11836205; doi:10.1038/s41598-025-86914-y)
Supplement: Supplementary file 1 — Supplementary Material 1 [file 41598_2025_86914_MOESM1_ESM.docx]

**Supplemental Figures**

**
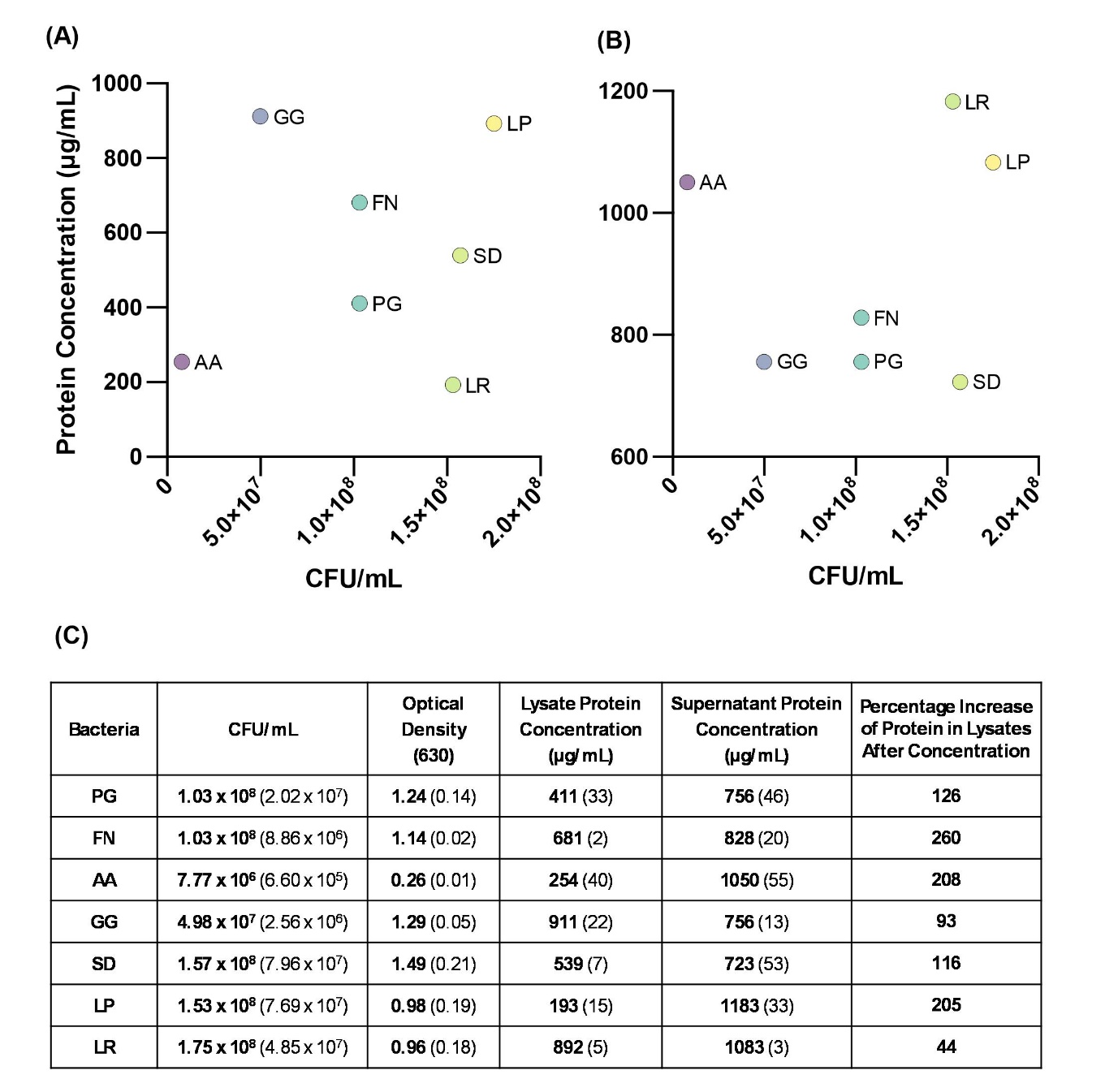
**

Supplemental Figure 1. The relationship between colony forming units per mL (CFU/mL) and optical density (OD), as well as lysate and supernatant concentration after 48h of culture. Graphs show the protein concentration of (A) lysates or (B) supernatants compared to CFU/mL (CFU/mL, n=3-6; protein concentrations, n=2-4). (C) A table demonstrating the mean values (and standard deviation in brackets) for CFU/mL (n=3-6), OD (n=3-6), lysate and supernatant protein concentration (n=2-4). The final column also shows the mean percentage increase in protein concentration of lysates after preparation with 3kDa protein concentrators (n=2-3). Porphyromonas gingivalis (PG), Fusobacterium nucleatum (FN), Aggregatibacter actinomycetemcomitans (AA), Lactobacillus rhamnosus (GG), Lactobacillus rhamnosus (SD), Lactobacillus plantarum (LP), Lactobacillus reuteri (LR).

**
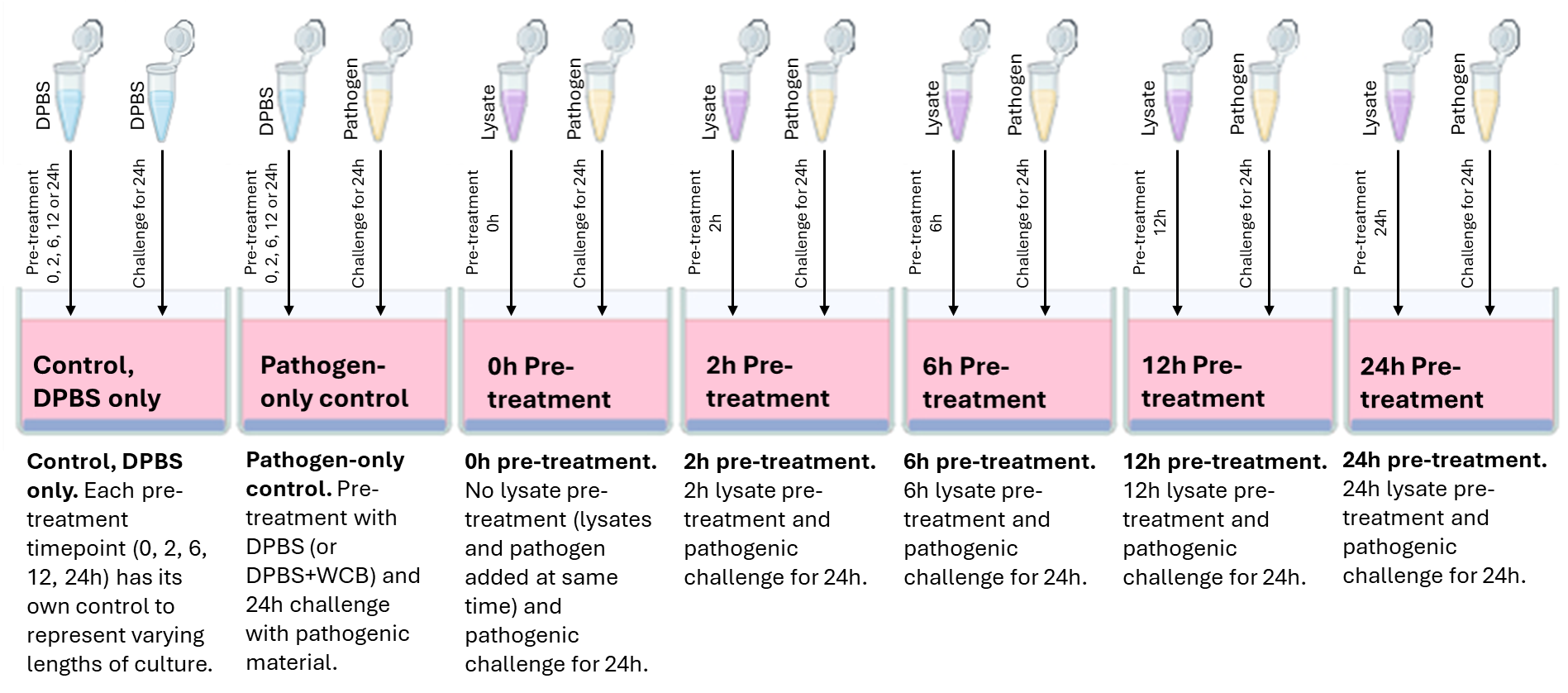
**

Supplemental Figure 2. A diagram illustrating the controls used in probiotic pre-treatment experiments to assess the protective effects of Lactobacillus lysates against periodontal pathogens, evaluated through cell viability counts. Made in Biorender.com.


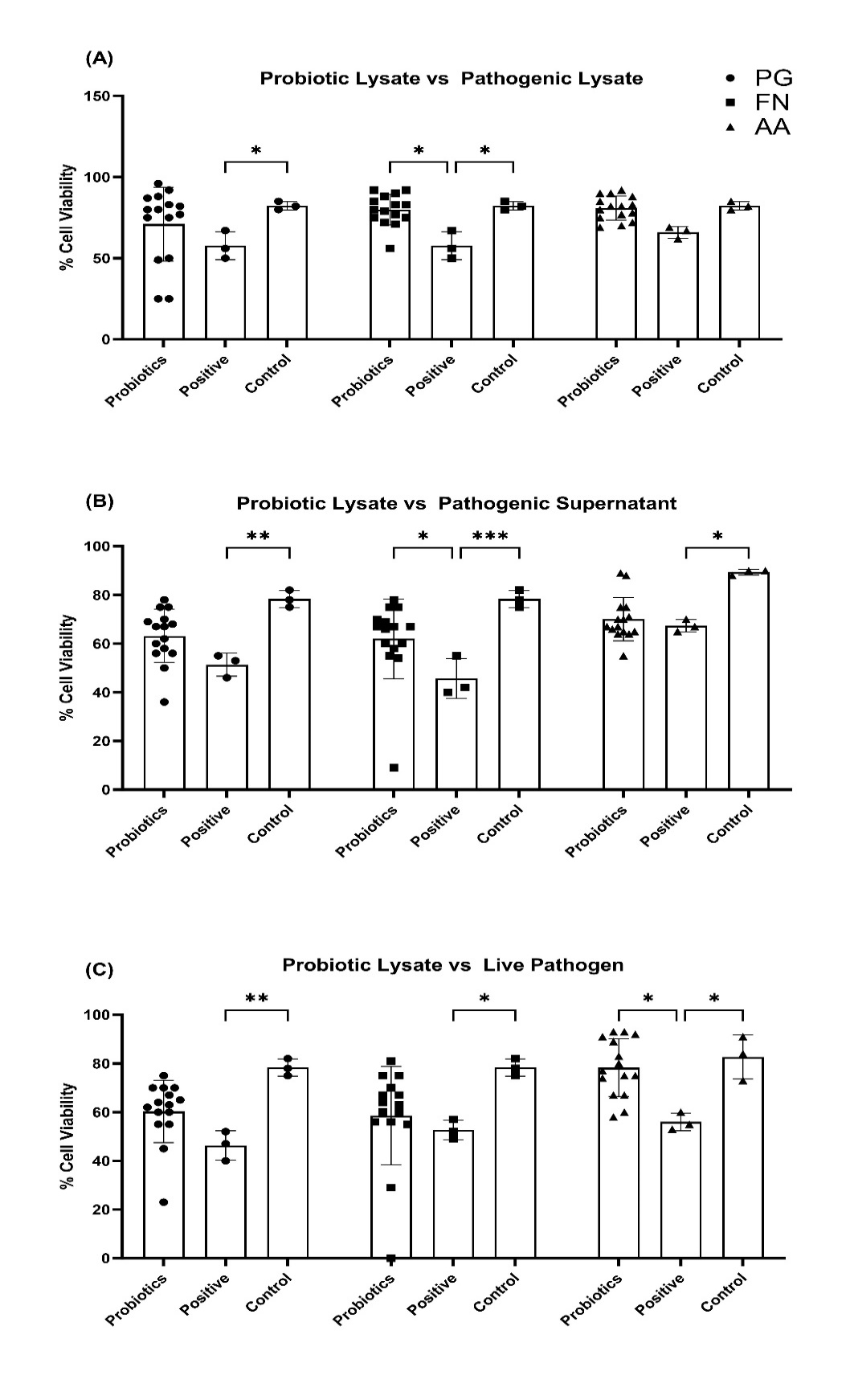


Supplemental Figure 3. Cell viability of TR146 cells after 24h pre-treatment with probiotic lysates (250μg/mL) and challenged for 24h with pathogenic lysate (A), supernatant (B) and live pathogen (C). Pathogenic lysates and supernatants were added at (250μg/mL), live pathogen was added at 10^4^ CFU/mL. Porphyromonas gingivalis (PG), Fusobacterium nucleatum (FN), Aggregatibacter actinomycetemcomitans (AA). Probiotics contains averaged results from treatments with GG, SD LP and LR. Control represents TR146 cells where DPBS was added (A and C) or DPBS and WC broth (B) was added in place of treatment. Positive represents TR146 cells which did not receive probiotic pre-treatment but were challenged with pathogen. Bars represent the mean+/- SD (n=15 for probiotics, n=3 for positive and control). Two-way ANOVA *(p<0.05), **(p<0.01), ***(p<0.001), ****(p<0.0001).


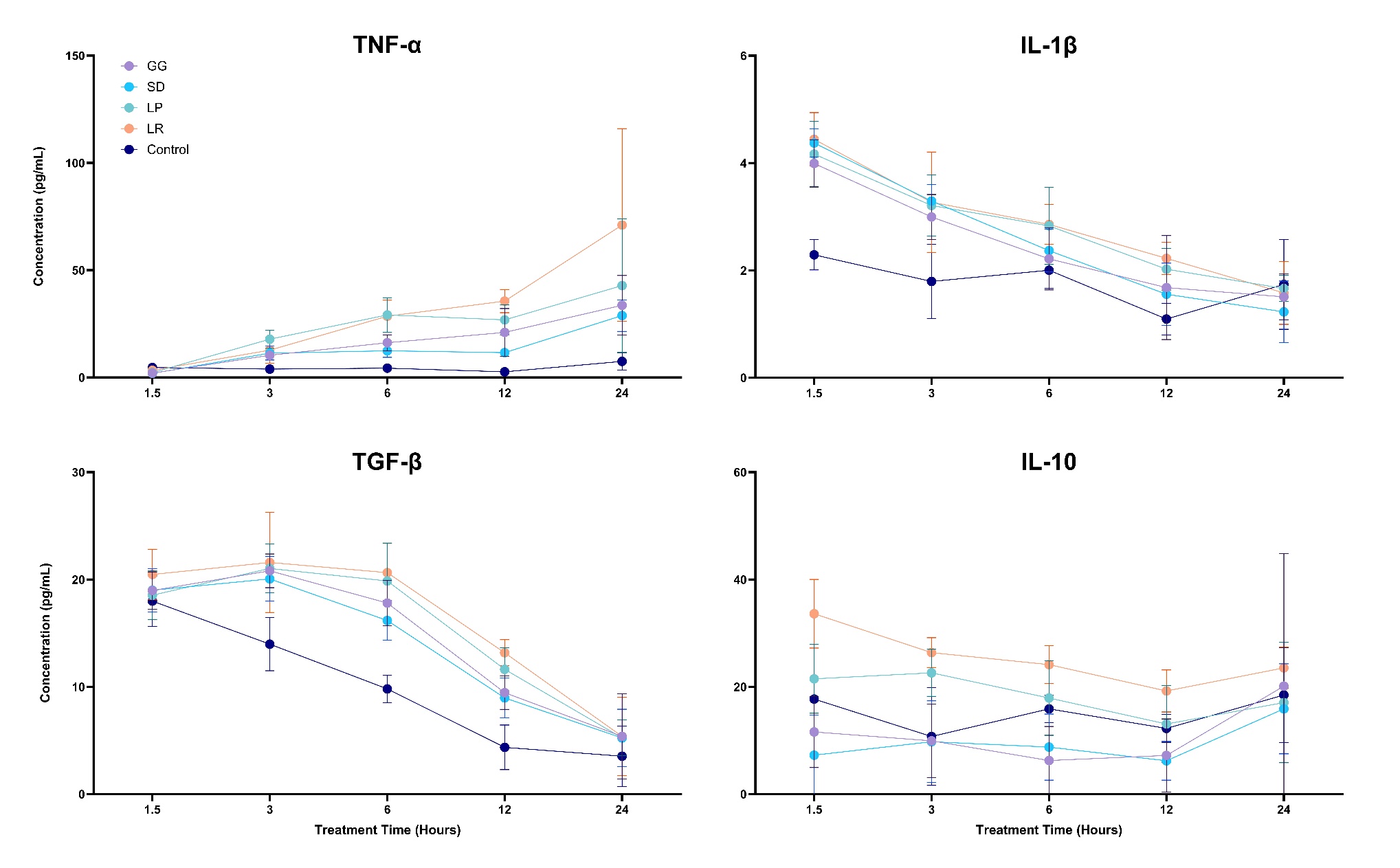


Supplemental Figure 4. Cytokine expression of TR146 cells treated with probiotic lysates (250 μg/mL) for 1.5, 3, 6, 12 and 24h. Lactobacillus rhamnosus (GG) n=12, Lactobacillus rhamnosus (SD), Lactobacillus plantarum (LP), Lactobacillus reuteri (LR) n=6. Control refers to cells where DPBS was added in place of treatment n=4. Bars represent the mean+/- SD.


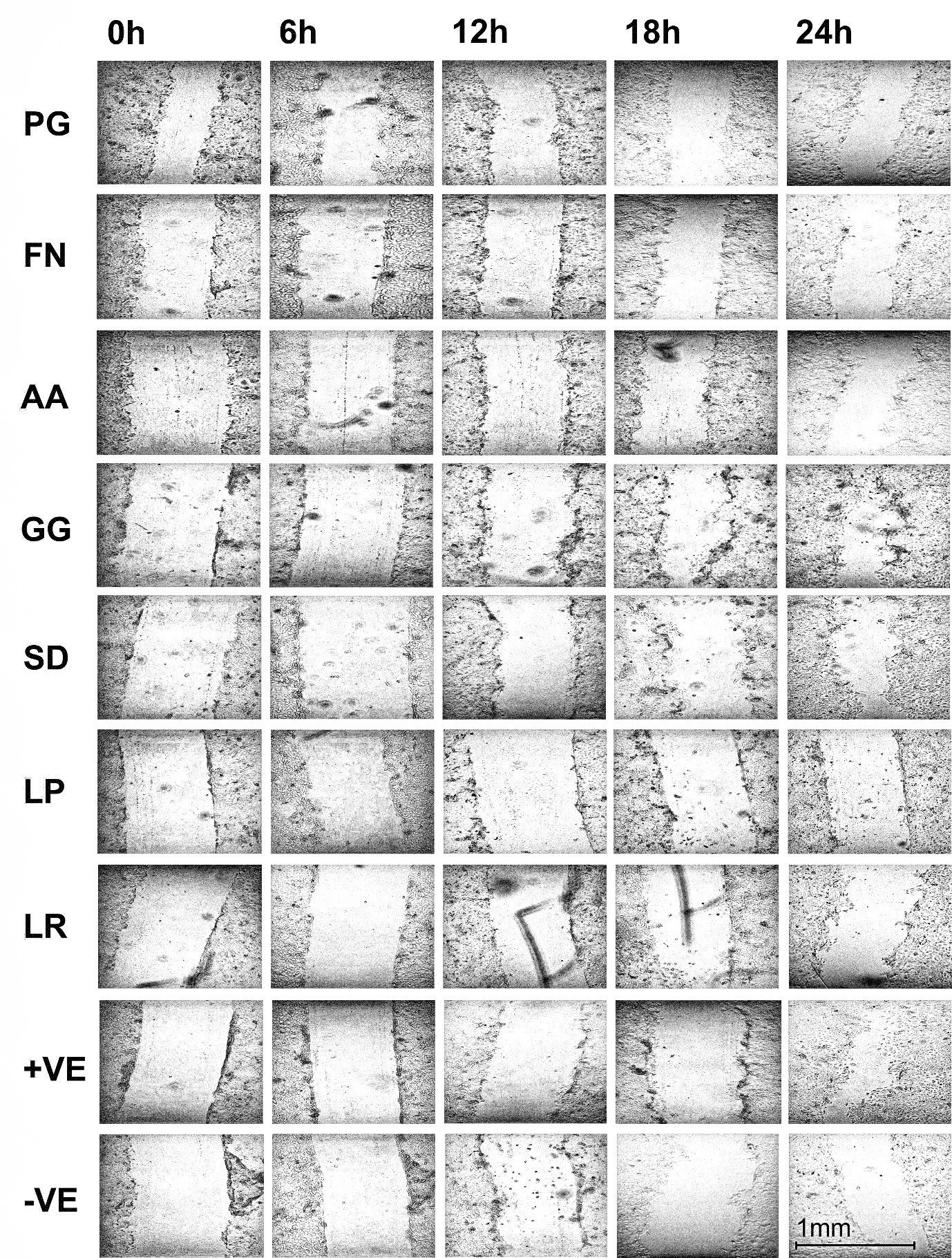


Supplemental Figure 5. Representative scratch images after 24h time-course treatment with pathogenic or probiotic lysates (250μg/mL). Porphyromonas gingivalis (PG), Fusobacterium nucleatum (FN), Aggregatibacter actinomycetemcomitans (AA), Lactobacillus rhamnosus (GG), Lactobacillus rhamnosus (SD), Lactobacillus plantarum (LP), Lactobacillus reuteri (LR). -ve represents cells treated with DPBS in place of lysate. +ve represents cells treated with 1% HKGS.

**Supplemental Table 1:**

Summary of statistically significant p-values from cytokine analysis. All statistics are compared to the control except comparisons between probiotics (e.g. GG vs LP). Two-Way ANOVA with a Dunnett’s posthoc test was performed for statistics compared to the control, Two-Way ANOVA with a Tukey’s posthoc test was used for comparison between probiotics. *(p<0.05), **(p<0.01), ***(p<0.001), ****(p<0.0001).

| **Cytokine** | **Probiotic Lysate** | **Treatment  Time (hours)** | **P-value  Summary** | **Adjusted P-value** |
| --- | --- | --- | --- | --- |
| **IL-8** | **GG** | 12 | *** | 0.0001 |
|  |  | 24 | **** | <0.0001 |
|  | **SD** | 24 | **** | <0.0001 |
|  | **LP** | 6 | ** | 0.0039 |
|  |  | 12 | **** | <0.0001 |
|  |  | 24 | **** | <0.0001 |
|  | **LR** | 12 | **** | <0.0001 |
|  |  | 24 | **** | <0.0001 |
|  | **SD vs LP** | 12 | ** | 0.0091 |
|  | **SD vs LR** | 12 | ** | 0.0033 |
| **IL-6** | **GG** | 12 | ** | 0.0092 |
|  |  | 24 | *** | 0.0005 |
|  | **SD** | 12 | * | 0.0187 |
|  |  | 24 | ** | 0.0014 |
|  | **LP** | 6 | ** | 0.0053 |
|  |  | 12 | **** | <0.0001 |
|  |  | 24 | **** | <0.0001 |
|  | **LR** | 12 | ** | 0.0012 |
|  |  | 24 | **** | <0.0001 |
|  | **GG vs LP** | 6 | * | 0.0171 |
| **IP-10** | **GG** | 24 | * | 0.0157 |
|  | **LP** | 12 | * | 0.014 |
|  | **LR** | 24 | * | 0.0371 |
| **IL-1α** | **GG** | 1.5 | * | 0.0182 |
|  |  | 3 | ** | 0.0017 |
|  |  | 24 | ** | 0.0046 |
|  | **SD** | 1.5 | * | 0.0126 |
|  |  | 3 | *** | 0.0009 |
|  |  | 24 | * | 0.0278 |
|  | **LP** | 1.5 | * | 0.0148 |
|  |  | 3 | *** | 0.0005 |
|  |  | 6 | * | 0.043 |
|  |  | 12 | * | 0.0123 |
|  |  | 24 | ** | 0.0027 |
|  | **LR** | 1.5 | *** | 0.0005 |
|  |  | 3 | * | 0.0205 |
|  |  | 6 | * | 0.0214 |
|  |  | 12 | ** | 0.0075 |
|  |  | 24 | ** | 0.0043 |
| **TNF-α** | **GG** | 12 | * | 0.0284 |
|  |  | 24 | *** | 0.0009 |
|  | **SD** | 24 | * | 0.0215 |
|  | **LP** | 6 | ** | 0.0058 |
|  |  | 12 | ** | 0.0074 |
|  |  | 24 | **** | <0.0001 |
|  | **LR** | 6 | ** | 0.0073 |
|  |  | 12 | *** | 0.0001 |
|  |  | 24 | **** | <0.0001 |
|  | **GG vs LR** | 24 | **** | <0.0001 |
|  | **SD vs LR** | 12 | ** | 0.0064 |
|  |  | 24 | **** | <0.0001 |
| **IL-10** | **GG vs LR** | 1.5 | **** | <0.0001 |
|  |  | 3 | * | 0.0121 |
|  |  | 6 | ** | 0.0022 |
|  | **SD vs LR** | 1.5 | **** | <0.0001 |
|  |  | 3 | * | 0.035 |
|  |  | 6 | * | 0.0443 |
| **IL-1β** | **GG** | 1.5 | **** | <0.0001 |
|  |  | 3 | *** | 0.0009 |
|  | **SD** | 1.5 | **** | <0.0001 |
|  |  | 3 | *** | 0.0002 |
|  | **LP** | 1.5 | **** | <0.0001 |
|  |  | 3 | *** | 0.0004 |
|  |  | 12 | * | 0.0326 |
|  | **LR** | 1.5 | **** | <0.0001 |
|  |  | 3 | *** | 0.0002 |
|  |  | 12 | ** | 0.0066 |
| **TGF-β** | **GG** | 3 | **** | <0.0001 |
|  |  | 6 | **** | <0.0001 |
|  |  | 12 | ** | 0.0018 |
|  | **SD** | 3 | *** | 0.0008 |
|  |  | 6 | *** | 0.0004 |
|  |  | 12 | * | 0.0153 |
|  | **LP** | 3 | **** | <0.0001 |
|  |  | 6 | **** | <0.0001 |
|  |  | 12 | **** | <0.0001 |
|  | **LR** | 3 | **** | <0.0001 |
|  |  | 6 | **** | <0.0001 |
|  |  | 12 | **** | <0.0001 |
|  | **GG vs LR** | 12 | * | 0.0259 |
|  | **SD vs LR** | 6 | * | 0.0186 |
|  |  | 12 | * | 0.0308 |
