# Supplementary figures and images for: Lactobacillus lysates protect oral epithelial cells from pathogen-associated damage, increase secretion of pro-inflammatory cytokines and enhance barrier integrity
Source: Sci Rep. 2025 Feb 18;15:5894. doi: 10.1038/s41598-025-86914-y (PMC11836205; doi:10.1038/s41598-025-86914-y)

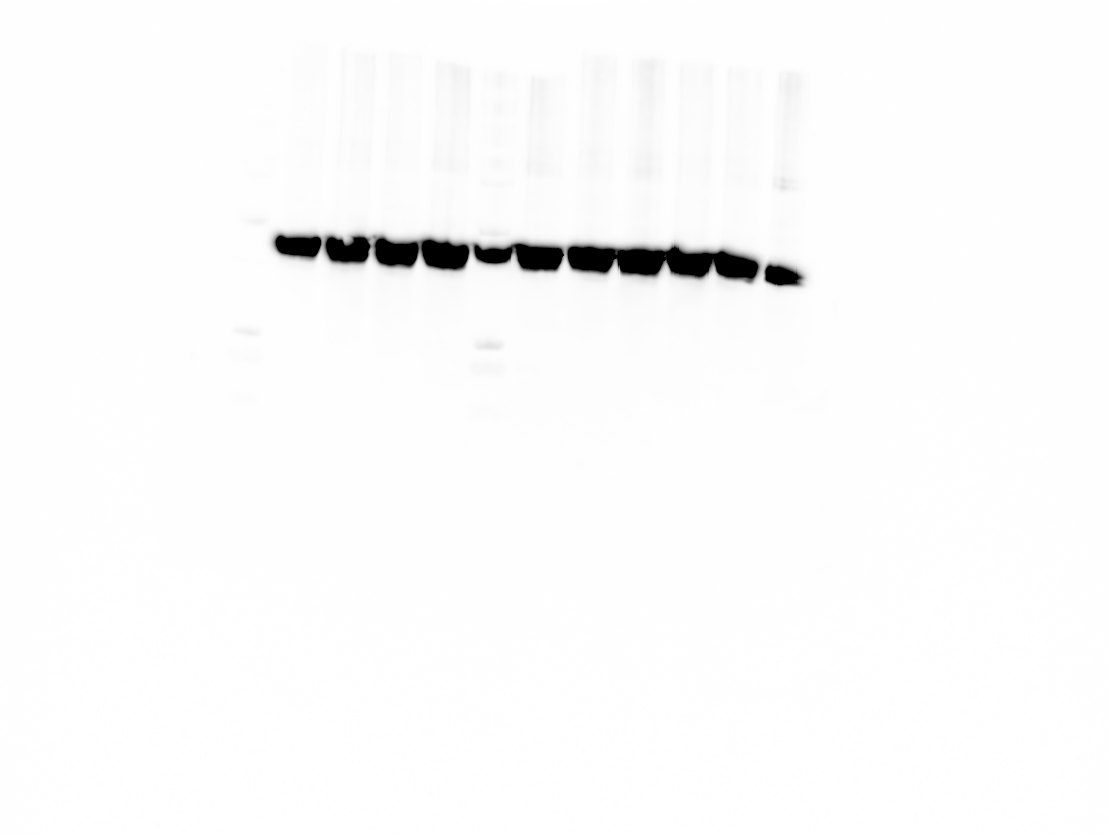

Supplement: Supplementary file 2 — Supplementary Material 2 [file 41598_2025_86914_MOESM2_ESM.tif]

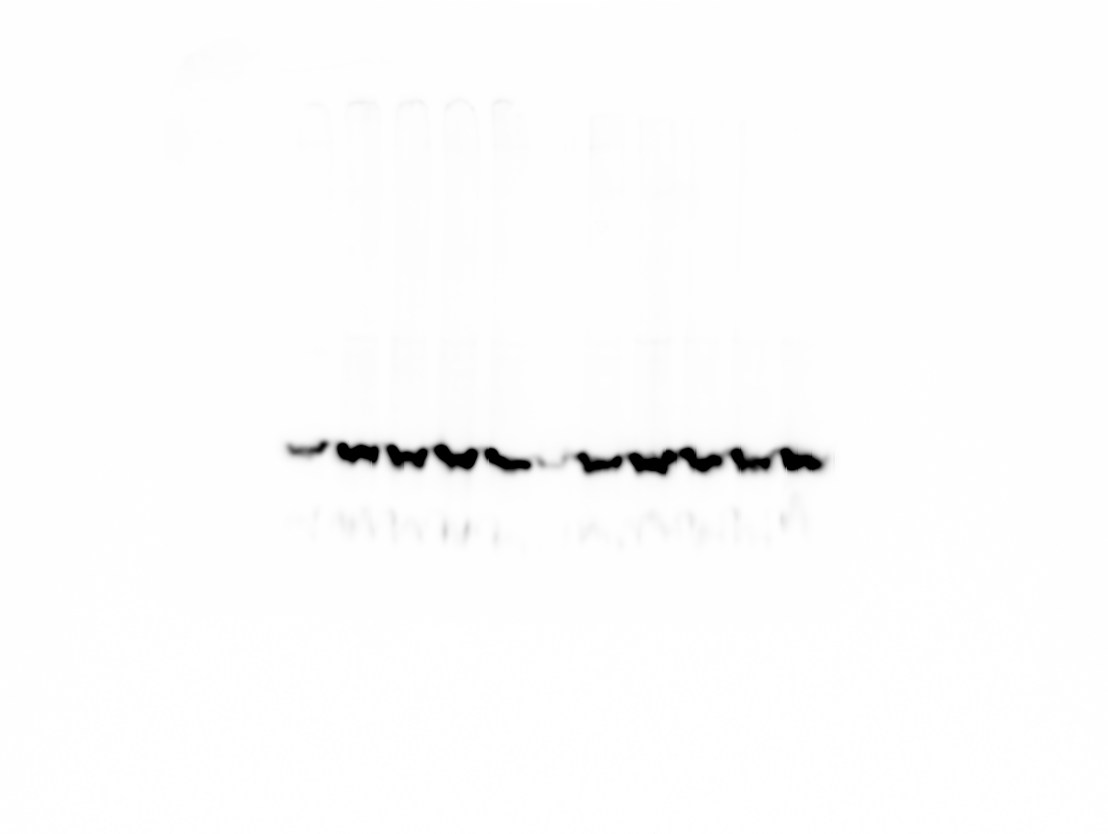

Supplement: Supplementary file 3 — Supplementary Material 3 [file 41598_2025_86914_MOESM3_ESM.tif]

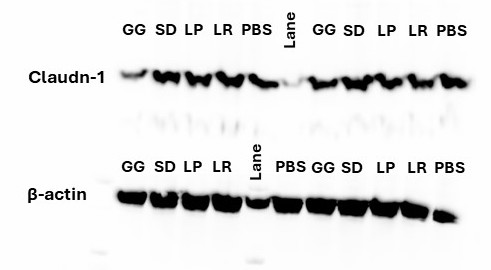

Supplement: Supplementary file 4 — Supplementary Material 4 [file 41598_2025_86914_MOESM4_ESM.jpg]
